# Thermographic imaging of mouse across circadian time reveals body surface temperature elevation associated with non-locomotor body movements

**DOI:** 10.1371/journal.pone.0252447

**Published:** 2021-05-28

**Authors:** Hiroyuki Shimatani, Yuichi Inoue, Yota Maekawa, Takahito Miyake, Yoshiaki Yamaguchi, Masao Doi

**Affiliations:** Department of Systems Biology, Graduate School of Pharmaceutical Sciences, Kyoto University, Kyoto, Japan; McGill University, CANADA

## Abstract

Circadian clocks orchestrate multiple different physiological rhythms in a well-synchronized manner. However, how these separate rhythms are interconnected is not exactly understood. Here, we developed a method that allows for the real-time simultaneous measurement of locomotor activity and body temperature of mice using infrared video camera imaging. As expected from the literature, temporal profiles of body temperature and locomotor activity were positively correlated with each other. Basically, body temperatures were high when animals were in locomotion. However, interestingly, increases in body temperature were not always associated with the appearance of locomotor activity. Video imaging revealed that mice exhibit non-locomotor activities such as grooming and postural adjustments, which alone induce considerable elevation of body temperature. Noticeably, non-locomotor movements always preceded the initiation of locomotor activity. Nevertheless, non-locomotor movements were not always accompanied by locomotor movements, suggesting that non-locomotor movements provide a mechanism of thermoregulation independent of locomotor activity. In addition, in the current study, we also report the development of a machine learning-based recording method for the detection of circadian feeding and drinking behaviors of mice. Our data illustrate the potential utility of thermal video imaging in the investigation of different physiological rhythms.

## Introduction

Locomotor activity rhythm (LAR) and body temperature rhythm (BTR) have been used to detect circadian rhythms in living animals because they are both robust and easy to measure. However, there are unfilled opportunities for the close analysis of LAR and BTR. LAR is usually detected by either an infrared sensor or a running wheel [1–3]. However, these methods cannot detect non-locomotor movements (i.e., activities that do not involve movement from place to place [4]), including feeding, drinking, grooming, and postural adjustments. For the time-series quantification of body temperature, measurements are usually performed by implanting a thermal sensor into the abdominal cavity of animals [5–7]. However, to conduct such measurements, surgery is necessary. Moreover, a sensor device is often large for small laboratory animals such as mice, making these experiments highly invasive. Additionally, long-term measurement of BTR with high time resolution over several days has been difficult because of the limited data storage performance of thermal sensor devices. These limitations have hampered the elucidation of how LAR and BTR are temporally correlated to each other.

To solve these technical problems, we developed a method for the simultaneous measurement of body movement (BM) and body surface temperature (BST). We captured thermographic video images of mice and calculated the BST and BM. These measurements identified increased BST prior to the initiation of locomotor activity. In this pre-locomotion period, mice exhibited non-locomotor movements, suggesting that non-locomotor movements were necessary for mice to “warm up” before the initiation of locomotor activity. In addition to these findings, we systemically characterized the inter-relationship between the appearance of locomotor activity and non-locomotor activity. We also developed a machine learning-based method for the extraction of feeding/drinking behaviors of animal. We discuss the potential utility of thermal video imaging in the investigation of different physiological rhythms.

## Materials and methods

### Animals

C57BL/6J mice were purchased from CREA Japan Inc. Only male mice were used to avoid confounding effects of the estrous cycle, which affects body temperature rhythm in females. Throughout the experiments animals were housed under constant ambient temperature of 22 ± 0.5°C, which is below the thermoneutral point of mice [8, 9]. Before the experiments, animals were acclimated for at least two weeks in a 12-h light:12-h dark cycle. All animal studies were performed using protocols approved by the Animal Experimentation Committee of Kyoto University.

### Infrared imaging

For thermographic analysis, mice were housed individually in experimental cages and monitored using a thermal imaging camera (Tau 2, FLIR Systems). A Camera Link frame grabber (ThermalCapture Grabber USB, TEAX Technology) was installed on the camera to extract digital images. To clearly detect the body surface temperature of mice, the back hair was removed with a razor. Thermal video images of the entire cage were recorded at a resolution of 640 × 512 pixels. The area of the mouse was roughly 4000 pixels. Thermal images were collected at a frequency of 30 Hz. To reduce computation time, video recordings were down-sampled to two frames per second for all analyses.

### Calculation of BST from thermal images

We defined BST as the 200th highest value for each frame. To correct for internal measurement errors, we used room temperature (median value of each frame) as the reference point. For each frame, the magnitude of the internal error was calculated as the difference between the raw and 600 sec (1200 point) moving average value of room temperature. The BST was then corrected by subtracting the calculated internal error. BST data were smoothed once with the 120 point moving average because BST can fluctuate greatly depending on the posture of mice.

### Calculation of BM from thermal images

To automatically find the positions of mice from thermal images, we first binarized thermal images using the 4000th highest values of frames as thresholds. Then, we clustered pixels of binary images using DBSCAN (RAPIDS cuML library, eps = 2.24, min_samples = 18), and the largest cluster was defined as the area of the mouse. The centroid of the mouse area was used as a reference point. Body movement (BM) was defined as the distance between the reference points between frames. Then, we used 60 s (120 point) moving median of BM (mBM) for the classification of behavior into three different categories, locomotor movement (mBM > 10), non-locomotor movement (0.8 < mBM ≤ 10) and resting (mBM ≤ 0.8). Transitions from resting to movement were extracted from data by selecting the events in which the animal’s behavior changed from resting (defined as 80% or more resting for 500 s) to movement (defined as continuous locomotor or non-locomotor movement for 300 s). Extracted events were hierarchically clustered using Ward’s method (scikit-learn library) using Euclidean distances among transition event data. For this cluster analysis, resting was set to 0, non-locomotor movement was set to 1, and locomotor movement was set to 2. For phase plot of non-locomotor movements that are not accompanied by the initiation of locomotion, events in which animals exhibit behavioral change from resting (defined as resting for ≥ 60 s) to continuous non-locomotor movement (also for ≥ 60 s) retuning to resting (≥ 60 s) were extracted, and these events were plotted in Rayleigh format using Oriana 4 software (Kovacs Computer Services, UK).

### Analysis of feeding and drinking behavior from thermal images

Automated detection of feeding and drinking was performed using DeepLabCut, an efficient method for pose estimation using deep neural networks [10]. We created and trained a DeepLabCut model for each animal using 150 video frames with manual labeling (nose, ears, neck, and back). The nose position of each mouse was then estimated for all images. If the estimated nose position was within the range of the food position for 2.5 s (i.e., five consecutive frames), we assessed it as feeding, and if the estimated nose position was within the range of the water bottle tip position for 1.5 s, we assessed it to be drinking.

### Statistical analysis

We used Welch’s analysis of variance (ANOVA) with *post-hoc* Games-Howell test to evaluate mean differences between different groups in Fig 3D. A value of *P* < 0.05 was taken as significant.

## Results

### BST measurements using thermal video imaging

**Fig 1:**
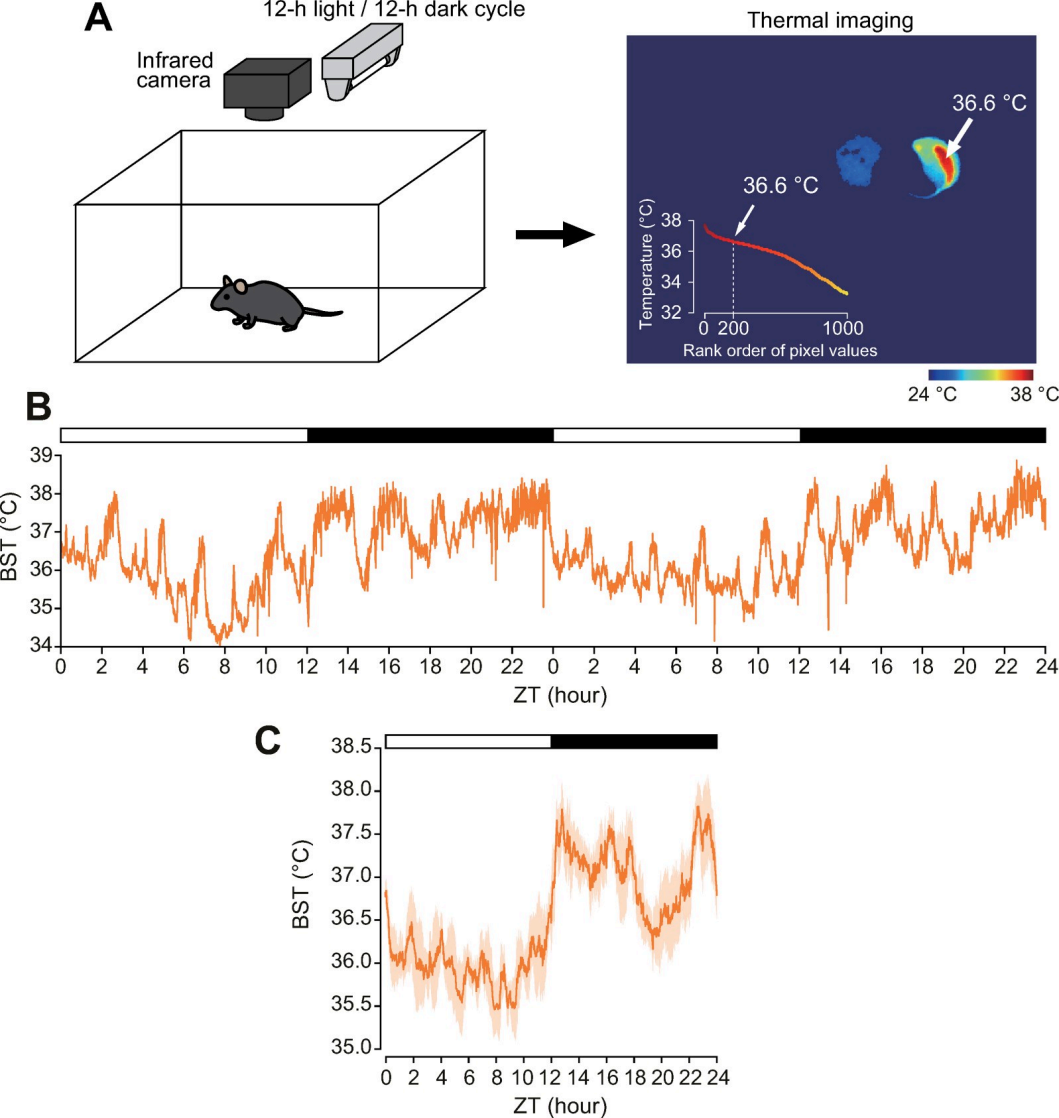
Infrared video camera-based monitoring of circadian body surface temperature. (**A**) Schematic representation of video imaging and representative thermography. The inset indicates temperatures of the top 1000 pixels of the mouse. Arrows indicate the 200th highest temperature. (**B**) Representative BST records of C57BL/6J mice under light/dark (LD) cycles for 2 days. BST was calculated every 0.5 s and smoothed once with a 120 point moving average. (**C**) Averages of 3 days recording of BST. Data are presented as mean ± standard error of mean (SEM); *n* = 4 mice.

To measure BST in a non-invasive way at high time resolution, we employed infrared camera-based thermal video imaging of living animals (Fig 1A). In previous reports, the highest body temperature of mice was defined as the BST [11, 12]. However, we noticed that the highest values tended to vary depending on the posture of the animal. Thus, in this study, we defined mouse BST as the 200th highest value (Fig 1A) since the body temperature slope was relatively constant around this point in our settings (see Fig 1A, inset). Raw BST values were smoothed once with a 120 point (60 s) moving average after internal measurement errors were corrected. The BST of C57BL/6J mice displayed a clear circadian rhythm of bimodal peaks, one in the early evening and again at dawn, with a deep trough at midnight (Fig 1B and 1C), which is in parallel with the temperature in the abdominal cavity of mice [6, 7].

### BM measurements using thermal video imaging

**Fig 2:**
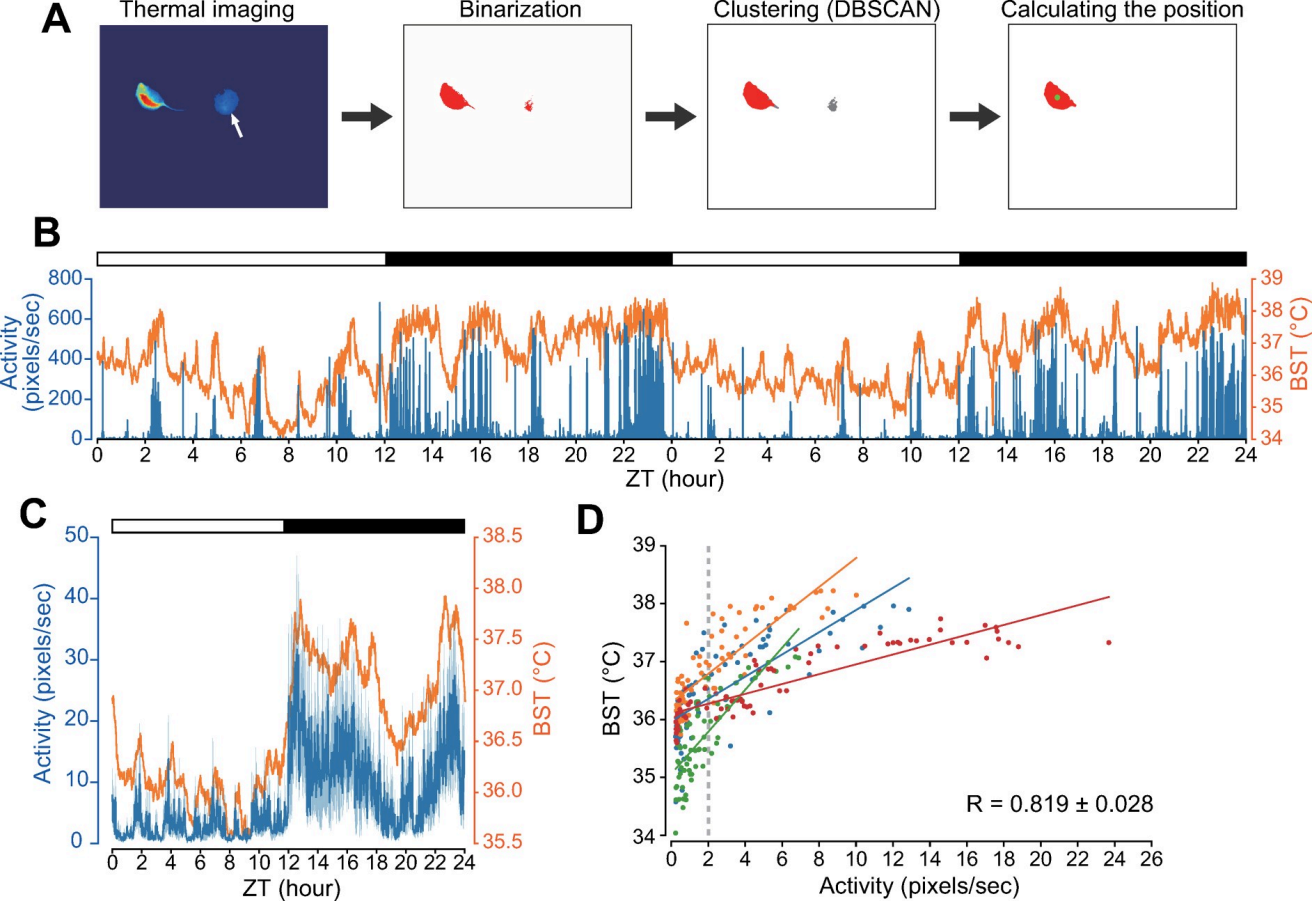
Infrared video camera-based monitoring of circadian body movement. (**A**) Representative imaging analysis for the determination of mouse position. The process involves binarization, clustering (DBSCAN), and calculating mouse position. A green dot on the red segments indicates the centroid of the area of mouse. A white arrow indicates the areas open but previously warmed by mouse. (**B**) Representative BST and BM records of C57BL/6J mice under LD for 2 days. (**C**) Averages of 3 days recording of BM and BST. Data are presented as mean ± SEM. *n* = 4 mice. (**D**) Scatter plots showing the relationship between BST and BM. Each data point represents averages of BST (y-axis) and BM (x-axis) for every 1 h. Each color represents an individual mouse (*n* = 4). Diagonal lines represent linear fitting of the data of each animal.

We next devised a method for quantifying body movement (BM) from thermal images. Thermal images exhibited relatively high temperatures not only in the location of the mouse but also in the areas where the mouse was previously located (Fig 2A, white arrow). Therefore, binarization by thresholding was insufficient for accurately calculating the location of the mouse. To solve this problem, in addition to binarization, a clustering method called DBSCAN [13] was used to distinguish the location of the mouse from regions of non-interest (Fig 2A). The centroid of the mouse area was used as a reference point. BM was defined as the distance between the reference points between frames. BM also displayed a clear circadian rhythm of bimodal peaks, one in the early evening and at dawn, with a deep trough at midnight, similar to locomotor activity measurements with the non-video-based recording system [1–3] (Fig 2B and 2C). As expected, a strong positive correlation was observed between BST and BM (R = 0.819 ± 0.128) (Fig 2D), which is consistent with previous reports [14, 15].

### Identification of BST elevation with non-locomotor movements before initiation of locomotor activity

**Fig 3:**
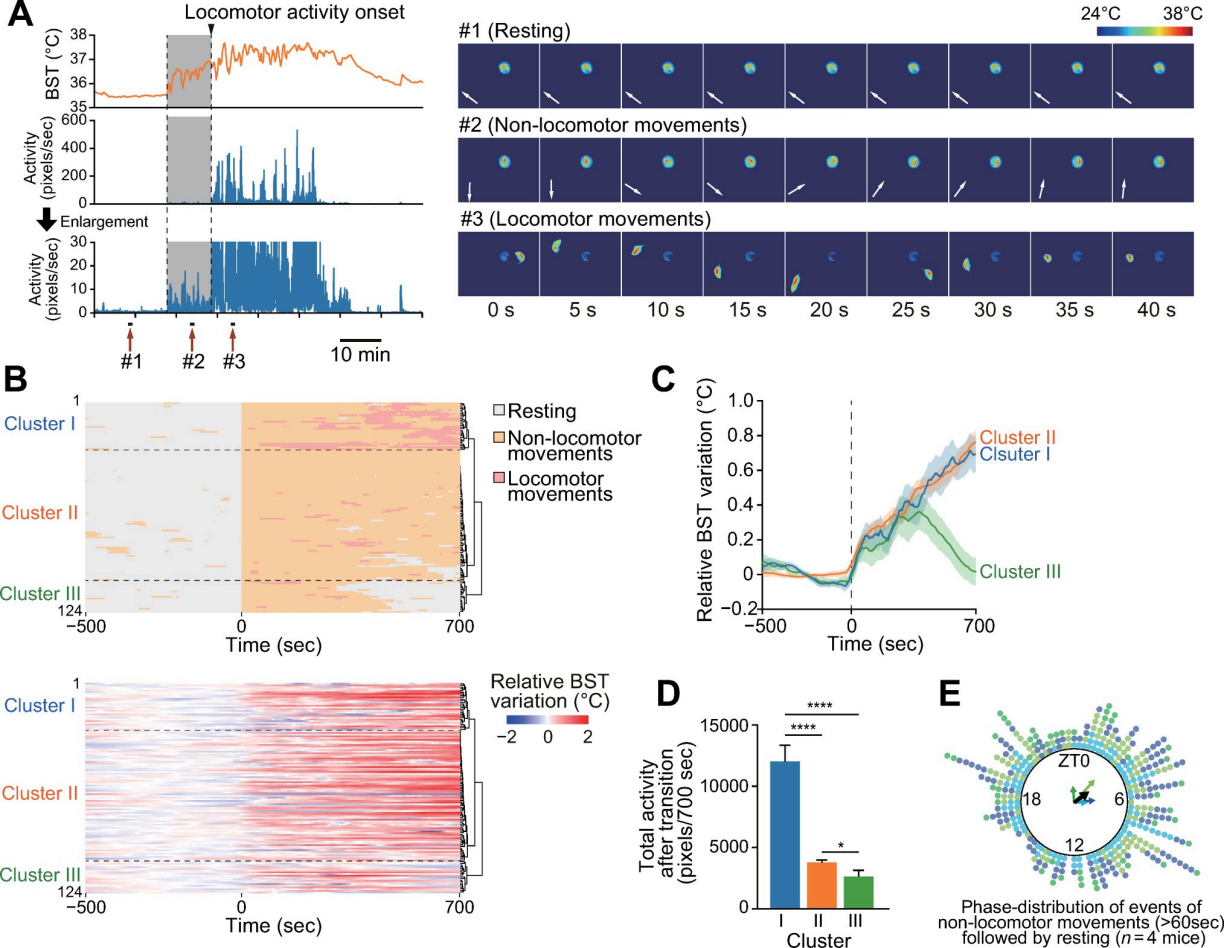
Identification of non-locomotor activities-associated BST elevation. (**A**) Representative BST and BM profiles under different BM states. A period of increase in BST prior to the initiation of locomotor activity is highlighted by a yellow background. Image data on the right show representative mouse positions under resting (#1), non-locomotor movements (#2), and locomotor movements (#3). White arrows on the images indicate the nose-tail directions of the mouse. (**B**) Alignments of all resting to movement transition events (*n* = 124 events for four mice). The upper and lower graphs indicate BM and BST data, respectively. For BST, average of 500 s before transition was set to 0. Time 0, time of transition. (**C**) Time course of average BST increase from resting-to-movement transitions in each cluster. (**D**) Total BM activity after the transition. *P < 0.05, ****P < 0.0001, Welch ANOVA *post-hoc* Games-Howell test. (**E**) Phase distribution of incidence of non-locomotor movements that are not accompanied by locomotor movements (*n* = 4 mice). Data from independent animals are color-coded. Non-locomotor activities that continued at least 60 s were selected. Arrows in the circle are the Rayleigh plot vector. The black vector indicates the average of all groups.

In addition to the gross correlation described above, we noticed that BST was not constantly low, rather variable, when BM values were low (Fig 2D). Thus, we next analyzed the temporal relationship between BST and BM when the mean BM value was lower than 2 pixels per sec. Interestingly, as shown in Fig 3A, this analysis led to the identification of increased BST prior to the initiation of locomotor activity. In this pre-locomotion period, BST was gradually increased without apparent locomotor activity (Fig 3A, middle), but appreciable BM values of about 1–10 pixels per second were captured within this period (Fig 3A, bottom), suggesting that BST elevation occurred in association with “non-locomotor” movement. To test this association in depth, we classified behaviors of mice into three distinct states according to BM using a 120 point (60 s) moving median of BM (mBM) value; resting (mBM ≤ 0.8), non-locomotor movement (0.8 < mBM ≤ 10), and locomotor movement (mBM > 10). Real-time video imaging confirmed that mice did not move during resting period (see #1 in Fig 3A and S1 Movie), that mice moved their bodies or groom themselves at the same location during non-locomotor movement (see #2 in Fig 3A and S1 Movie), and that mice moved around the cage during performance of locomotor movement (see #3 in Fig 3A and S1 Movie). To analyze the relationship between BST and BM, we extracted all transition events from resting to movement from all video data (*n* = 124 events from four mice; Fig 3B and S2 Movie). Overall, BST showed gradual increase at the transition from resting to movement (Fig 3B). To perform a systematic analysis, we conducted unsupervised hierarchical clustering analysis of BM states around the transitions (from −500 s to +700 s) and found that the patterns of emergent order of BM states could be separated into three distinct clusters: cluster I (resting → non-locomotor movement → locomotor movement), cluster II (resting → non-locomotor movement), and cluster III (resting → non-locomotor movement → resting). Noticeably, there were no events showing an immediate transition from resting to locomotor movement, indicating that non-locomotor movements always precede locomotor movements. Nevertheless, non-locomotor movements are not always accompanied by locomotor movement. These behavioral patterns are consistent with a previous report [4]. As expected, BST decreased after the transition from non-locomotor movement to resting (Fig 3C; cluster III). Interestingly, elevated BST values in clusters I and II were almost comparable to each other, while total BM activity after the transition of cluster I was significantly higher than that of cluster II (Fig 3D). These results suggest that non-locomotor movement is an independent mechanism of thermogenesis, comparable to active movement. We also examined the phase distribution of the occurrence of non-locomotor movements that are not associated with the initiation of locomotor activity. To this end, events in which mice show non-locomotor activities for more than 60 s were selected, and we found that these events were mainly observed during the light phase (Fig 3E).

### Analysis of feeding and drinking behavior from video imaging

**Fig 4:**
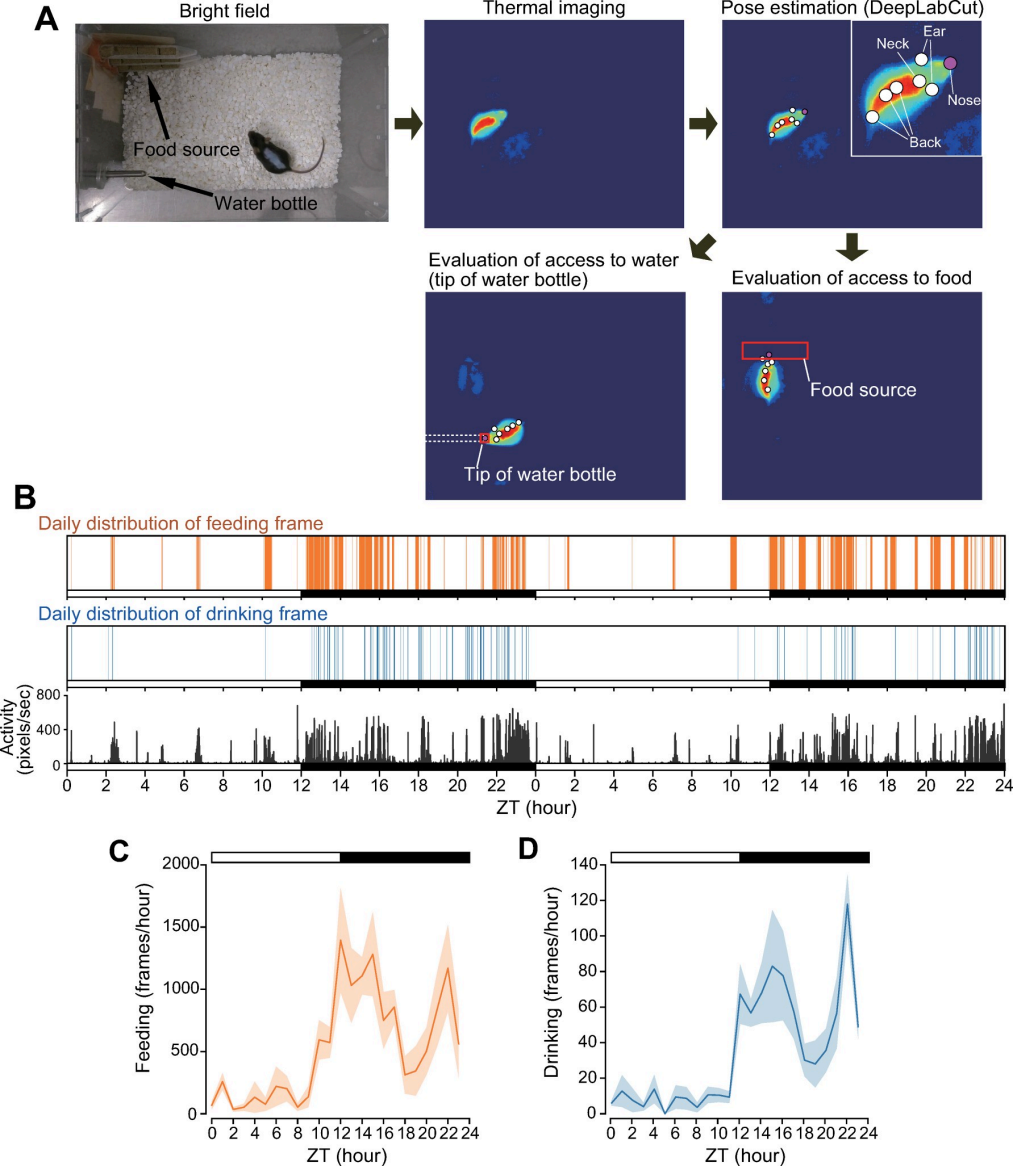
Infrared video camera-based monitoring of circadian feeding and drinking behavior. (**A**) Representative imaging analysis for the determination of feeding and drinking behavior. The process involves thermal imaging, pose estimation (DeepLabCut), and evaluation of access to food or water (tip of water bottle). (**B**) Representative BST, BM, feeding, and drinking records of C57BL/6J mice under LD cycle for 2 days. (**C**, **D**) Averages of 3 days recording of feeding (**C**) and drinking (**D**). Data are presented as mean ± SEM. *n* = 4 mice.

In addition to LAR and BTR, various other physiological phenomena are controlled by the internal circadian clock [16–18]. Thus, we developed a system to detect drinking and feeding behavior using video imaging. To do so, we determined the nose position of the animal using the DeepLabCut algorithm, which allows for efficient and accurate pose estimation [10]. First, we randomly extracted frames and labeled them by body parts (nose, neck, ears, and back) (Fig 4A). Next, we trained the DeepLabCut models and estimated the positions of the body parts for all images. Feeding and drinking were assessed by whether the estimated nose position overlapped with the area of the food source and the tip of the water bottle, respectively (Fig 4A and S3 and S4 Movies). The frequency of feeding and drinking displayed clear circadian rhythms of bimodal peaks, one in the early evening and at dawn, with a deep trough at midnight (Fig 4B–4D), which is consistent with previous reports [18–21].

## Discussion

By measuring BM and BST with an infrared camera, we visualized previously unknown BST elevation in mice that occurred in close association with non-locomotor activity. Crucially, active locomotor movements were always preceded by non-locomotor activity-associated BST elevation, suggesting that non-locomotor activity and its associated body temperature elevation may be necessary for mice to initiate locomotor activity. Interestingly, non-locomotor movements were not always accompanied by locomotor activity, suggesting that non-locomotor activity-associated BST elevation may play a role independent of locomotion. Particularly, non-locomotor movements that are not linked to active locomotion were mainly observed during the light phase, in which mice are primarily resting. Although this may simply reflect the fact that during the light phase waking events from sleep do not lead to full arousal in mice, it is tempting to speculate that non-locomotor movement may play a role in thermogenesis during resting time to maintain homeostasis of body temperature.

Although non-locomotor movements such as grooming and postural adjustments appear to be important for body temperature regulation, we should mention that non-locomotor movements cannot explain all BST variations observed at the daytime/resting phase. Indeed, small but non-negligible BST variations were displayed by mice at rest without appreciable non-locomotor movements, suggesting the presence of BM-independent body temperature regulation. One possible BM-independent mechanism may involve the brown adipose tissue (BAT). BAT produces heat in response to sympathetic activation without physical activity [22]. Another possible mechanism is skeletal muscle-mediated thermogenesis [23, 24]. BM-independent BST fluctuation may be caused by skeletal muscle activity which cannot be captured by the video-based method. Additionally, skeletal muscle has recently been shown to possess a heat-producing mechanism that does not involve muscle contraction [23], which may also contribute to BM-independent BST elevation.

Previous human studies reported that not only high-intensity exercise, such as sports and fitness-related activities, but also activities of daily living, such as sitting, standing, walking, and fidgeting increase energy expenditure and mediate resistance to weight gain with overfeeding [25, 26]. Interestingly, energy expenditure increases by ~50% with only fidgeting-like activities while seated [26]. In this study, we showed that non-locomotor activities of mice increased body temperature to levels almost comparable to or slightly lower than those observed during locomotor movement. Therefore, studying the role of non-locomotor movement-associated thermogenesis in mice may help to understand the impact of non-exercise activities on the daily regulation of energy homeostasis.

Circadian clocks orchestrate multiple physiological rhythms in a synchronized manner [16, 17], but little is known about how these separate rhythms are regulated cumulatively. In humans, body temperature fluctuates even when locomotor activity is restricted [27, 28], and BTR and locomotor activity rhythms can be experimentally dissociated, a phenomenon known as spontaneous internal desynchronization [29]. These data support the notion that the BTR and LAR are regulated separately [30]. In flies and mice, calcitonin receptors regulate BTR during the active phase without affecting LAR [7]. Additionally, it was recently revealed that the circadian clock neurons in the suprachiasmatic nucleus (SCN) that directly project to thirst neurons in the organum vasculosum lamina terminalis (OVLT) control water intake just before the resting period [19], suggesting that LAR and drinking rhythm are coordinately regulated. These recent findings highlight the increasing importance of simultaneous measurement of multiple physiological activity rhythms to elucidate coordinated circadian outputs. In this study, we showed that BST, BM, feeding, and drinking can be simultaneously measured using thermal video imaging and automated behavioral analyses. Thus, our method may be instrumental in understanding the interrelationship between these independent circadian behaviors in mice.

The method described here, however, has two drawbacks. First, for the sake of improving detection accuracy of body surface temperature, we shaved the back hair of animal. Circadian experiments are often run over several weeks to months; mice will have to be shaved at certain intervals, which could be an entraining stimulus. Secondly, in this study, BM was classified into three states, resting, non-locomotor movement, and locomotor movement, as previously reported [4]. However, the behavior of animal is more complex than classified. Non-locomotor movements are considered to include various heterogeneous behaviors, such as grooming, stretching, head dip, head raise, sniffing, and other small movements of limbs and/or torso. In addition, locomotor movements are also associated with a range of activities: walking, running, jumping, hanging, rearing, and digging. Mice also exhibit other behaviors such as nest-building, defecation, and urination. The power of video-based automated analysis of complex behaviors of mice has already been reported by previous pioneering works [31, 32]. The elucidation of temporal relationships between multiple behaviors and BST using thermal video imaging will be the subject of our future study.

## Supporting information

S1 Movie BST elevation with non-locomotor movements before initiation of locomotor activity. (MP4)

S2 Movie All video data in Fig 3B. (MP4)

S3 Movie Video camera-based monitoring of drinking behavior. (MP4)

S4 Movie Video camera-based monitoring of feeding behavior. (MP4)
